# Supplementary material for: Responsiveness of the Calf-Raise Senior test in community-dwelling older adults undergoing an exercise intervention program
Source: PLoS One. 2020 Apr 29;15(4):e0231556. doi: 10.1371/journal.pone.0231556 (PMC7190110; doi:10.1371/journal.pone.0231556)

Calf-raise Senior Test protocol

1. Initial positioning - barefoot, supporting the fingers at a wall (shoulder height) with the elbows slightly flexed, keeping the spine in neutral position, feet apart (hip width), and knees extended;
2. Amplitude definition – the participant raises his heels as high as possible, with knees fully extended, while the rater defines the range of movement using a square tool (used to support shelves) supported simultaneously at the top of his head and against the wall;
3. Execution – at the signal of the rater, the participant raises his heels vertically up to the maximum height possible, and then lowers it completely into the ground, performing the highest number of repetitions of this movement, in a pre-defined period of 30s. The participant should touch his head at the square whenever he reaches the maximum height, and also touch with the heels on the ground at the end of the cycle;
4. Rating - the number of correct elevations at the end of the 30 s

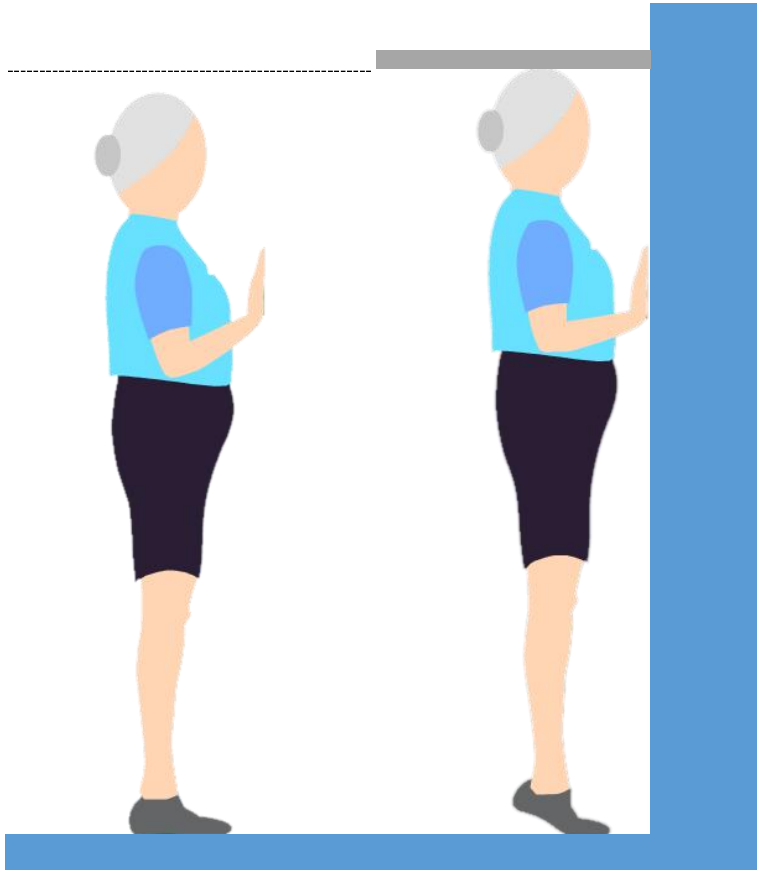

Supplement: S1 File — (ZIP) [file pone.0231556.s001.zip › Calf-raise senior test protocol.pdf]
